# MotifMap: integrative genome-wide maps of regulatory motif sites for model species

**DOI:** 10.1186/1471-2105-12-495

**Published:** 2011-12-30

**Authors:** Kenneth Daily, Vishal R Patel, Paul Rigor, Xiaohui Xie, Pierre Baldi

**Affiliations:** 1 Department of Computer Science, University of California Irvine, Irvine, CA 92697 USA; 2 Institute for Genomics and Bioinformatics, University of California Irvine, Irvine, CA 92697 USA; 3 Department of Developmental and Cell Biology, University of California Irvine, Irvine, CA 92697 USA

## Abstract

**Background:**

A central challenge of biology is to map and understand gene regulation on a genome-wide scale. For any given genome, only a small fraction of the regulatory elements embedded in the DNA sequence have been characterized, and there is great interest in developing computational methods to systematically map all these elements and understand their relationships. Such computational efforts, however, are significantly hindered by the overwhelming size of non-coding regions and the statistical variability and complex spatial organizations of regulatory elements and interactions. Genome-wide catalogs of regulatory elements for all model species simply do not yet exist.

**Results:**

The MotifMap system uses databases of transcription factor binding motifs, refined genome alignments, and a comparative genomic statistical approach to provide comprehensive maps of candidate regulatory elements encoded in the genomes of model species. The system is used to derive new genome-wide maps for yeast, fly, worm, mouse, and human. The human map contains 519,108 sites for 570 matrices with a False Discovery Rate of 0.1 or less. The new maps are assessed in several ways, for instance using high-throughput experimental ChIP-seq data and AUC statistics, providing strong evidence for their accuracy and coverage. The maps can be usefully integrated with many other kinds of omic data and are available at http://motifmap.igb.uci.edu/.

**Conclusions:**

MotifMap and its integration with other data provide a foundation for analyzing gene regulation on a genome-wide scale, and for automatically generating regulatory pathways and hypotheses. The power of this approach is demonstrated and discussed using the P53 apoptotic pathway and the Gli hedgehog pathways as examples.

## Background

A central challenge of biology is to map and understand gene regulation on a genome-wide scale. For any given genome, only a small fraction of the regulatory elements embedded in the DNA sequence have been characterized, and there is great interest in developing computational methods to systematically map all these elements and understand their relationships. Such computational efforts, however, are significantly hindered by the overwhelming size of non-coding regions and the statistical variability and complex spatial organizations of regulatory elements and interactions, especially in mammalian species.

While many gene-specific, condition-specific, and factor-specific resources for motif binding sites exist [1-4], it is perhaps surprising that genome-wide systematic catalogs of binding sites for most species do not. Past efforts have focused primarily on the yeast and fly genomes and with severe restrictions, for instance in terms of data (e.g. ChIP-seq only) or genomic regions (e.g. promoter only). The prototype MotifMap system [5] used an improved comparative genomics approach to provide one of the first genome-wide maps for the human genome and test its accuracy. This system, however, has several limitations including the direct use of coarse genome alignments for searching for binding sites leading to missed and incorrectly scored sites, and the unavailability of maps for other model species. Furthermore, while the available lists of transcription factors are not exhaustive, new information about transcription factors and regulatory interactions is continuously being produced and thus such maps must be periodically updated.

Here we describe improvements to the prototype methods that are used with a new whole-genome alignment and an expanded list of transcription factors to create a new, more comprehensive, map for the human genome. Furthermore, we apply the updated methodology to the genomes of other model organisms for which alignments and estimated phylogenetic trees are available, creating genome-wide maps for the yeast, worm, fly and mouse genomes.

At its core, MotifMap uses data from transcription factor binding motif databases, specifically JASPAR [6] and TRANSFAC [7]. For yeast and fly, we have supplemented the matrices available from JASPAR and TRANSFAC with those available from a number of publications (see Additional file 1 for a full list of the sources for each species). The binding matrices are used to search a reference genome for binding sites and produce three scores at each site. The first score is the Normalized Log-Odds (NLOD) score derived from the position weight matrix of the corresponding transcription factor. The second score is the Bayesian Branch Length Score (BBLS) to measure the degree of evolutionary conservation. Functional elements, such as those playing a regulatory role, often evolve more slowly than neutral sequences and can be detected by their higher level of conservation. MotifMap uses publicly available whole genome alignments and the corresponding phylogenetic trees to leverage the power of comparative genomics in order to eliminate false positive hits. The third score is the False Discovery Rate (FDR) estimated by using Monte Carlo methods. The three scores at each site are used, in combination with other filters, to generate genome-wide maps.

The quality of the maps is assessed and compared against our previous results [5] as well as other methods [8,9] in various ways, including comparison to experimental data, such as high-throughput ChIP-seq data. The maps provide a foundation for inferring regulatory networks and can be integrated with a variety of other heterogeneous and autonomous data sources.

## Methods

### Normalized Log-Odds score (NLOD)

Binding sites for each transcription factor are identified by scanning the genome sequence with a position weight matrix. We transform each original weight matrix into a log-odds matrix to account for the background frequency of the nucleotides across the genome. The log-odds score of a sequence is computed as

$$\text{LOD}\left(S\right)=\overset{|S|}{\underset{j=1}{\sum }}f\left(x\right)$$

Where

f(x)=log2(x)ifqij>ebi2cxe2c log(2)+cifqij≤ebi2c

where $x=\frac{q_{ij}}{b_{i}}$, the value *q_ij _*from the position weight matrix is the probability of observing nucleotide *i*({A, C, G, T}) at position *j *in a sequence *S *of length *|S|*, and *b_i _*is the probability of observing nucleotide *i *in the entire genome. For reasonable values of *q_ij _*corresponding to *x >*e2*^c^*, the function is simply equal to *log*_2_(*x*). However, for small values of *q_ij _*corresponding to *x *≤ e2*^c^*, the logarithm function can take large negative values. Traditionally, to avoid this problem, pseudocounts are added to the frequency matrices, in a heuristic and matrix-dependent fashion. The alternative approach proposed here lower bounds the values of each scoring matrix directly by replacing the log function around zero with a continuous linear approximation. In this work, we use *c *= -3.

The motif matching score is scaled to fall between 0 and 1 to yield the normalized log-odds score:

$$\text{NLOD}\left(x\right)=\frac{\text{LOD}\left(x\right)-y_{\text{min}}}{y_{\text{max}}-y_{\text{min}}}$$

where *y_max _*and *y_min _*are the maximum and minimum LOD scores that the matrix can achieve by using the most likely or least likely nucleotide at each position. A z-score is also derived from the NLOD score by estimating the mean and variance of the score of random sequences across the genome. For mammalian species, we use a *z*-score threshold of 4.27, corresponding to a *p*-value of 0.00001, to find a list of initial candidate sites across the reference genome. For yeast, fly, and worm, we use a lower threshold corresponding to a *z*-score between 2.57 and 3.72, or a *p*-value between 0.005 and 0.0001. Finally, we restrict the total number of binding sites by ordering the sites for each motif individually by their *z*-score, and keeping sites with a *z*-score at least as high as the *k^th ^*site. For our purposes, *k *= 100,000, as was done in the prototype version.

### Bayesian Branch Length Score (BBLS)

Many previous methods have shown that evolutionary conservation can be used to identify transcription factor binding sites [10-12]. An innovative aspect of the MotifMap system is how the degree of evolutionary conservation is assessed using the Bayesian Branch Length Score (BBLS) [5], which itself is an improvement over a previous score, the Branch Length Score (BLS) [13,14]. More precisely, given a multiple alignment of *N *species and their evolutionary tree, a transcription factor motif, and the genome coordinates of a candidate binding site, let *σ_i _*= 0 or 1 denote the presence or absence of the motif at the aligned location in the corresponding species *i*. The BLS is simply the total length of the branches associated with the most recent common ancestor of all the species for which *σ_i _*is set to 1. However, in reality *σ_i _*is not a binary variable but rather comes with a probability *p_i _*measuring the degree of confidence in whether the corresponding motif is present or not in species *i *at the corresponding location. Given a set of *N *aligned species, the BBLS takes into account this uncertainty by computing the *expected BLS *in the form:

(1) $$\begin{array}{ccc} BBLS & =E\left(BLS\right) & \\ & =\underset{\sigma_{1},\ldots ,\sigma_{N}}{\sum }P\left(\sigma_{1},\ldots ,\sigma_{N}\right)BLS\left(\sigma_{1},\ldots \sigma_{N}\right)\\ & =\underset{\sigma_{1},\ldots ,\sigma_{N}}{\sum }r_{1}\ldots r_{N}BLS\left(\sigma_{1},\ldots \sigma_{N}\right) \end{array}$$

Where

$$r_{i}=\left\{\begin{array}{cc} p_{i} & \text{if}\;\sigma_{\text{i}}\text{ = 1}\\ 1-p_{i} & \text{if}\;\sigma_{\text{i}}\text{ = 0} \end{array}\right)$$

The values of *p_i _*for the leaves of the tree are derived using the NLOD score described above. If the corresponding *z*-score is too low, *p_i _*is set to 0. An efficient dynamic programming approach, avoiding the addition of an exponential number of terms (Equation 1), has been derived [5], and a corresponding software implementation is available (see below).

### False Discovery Rate (FDR)

For every motif weight matrix, we generate control matrices by randomly shuffling the columns of the motif weight matrix. The shuffling is repeated up to 10,000 times so as to produce up to 10 control matrices. The shuffled matrices must be sufficiently different from the original one to be used as control matrices. In practice, we use a cutoff of 0.35 on the similarity measure computed by first taking the average correlation between columns over pairs of windows of length 8 in the original and permuted motif, then taking the maximum of these correlations over all pairs of windows, and then normalizing by the length of the motif. Only binding matrices are retained that: (1) are at least eight nucleotides long; and (2) can produce at least three sufficiently different shuffled versions for the Monte Carlo FDR procedure. In addition, for mammalian species, each shuffled matrix is restricted to have the same CG-dinucleotide frequency as the original matrix. The same motif searching procedure is used with each control matrix. The False Discovery Rate is computed as the median number of sites found using the shuffled matrices divided by the number of sites found for the real matrix at a particular (NLOD, BBLS) score combination or higher.

### Sequence alignments and modular design

**Figure 1:**
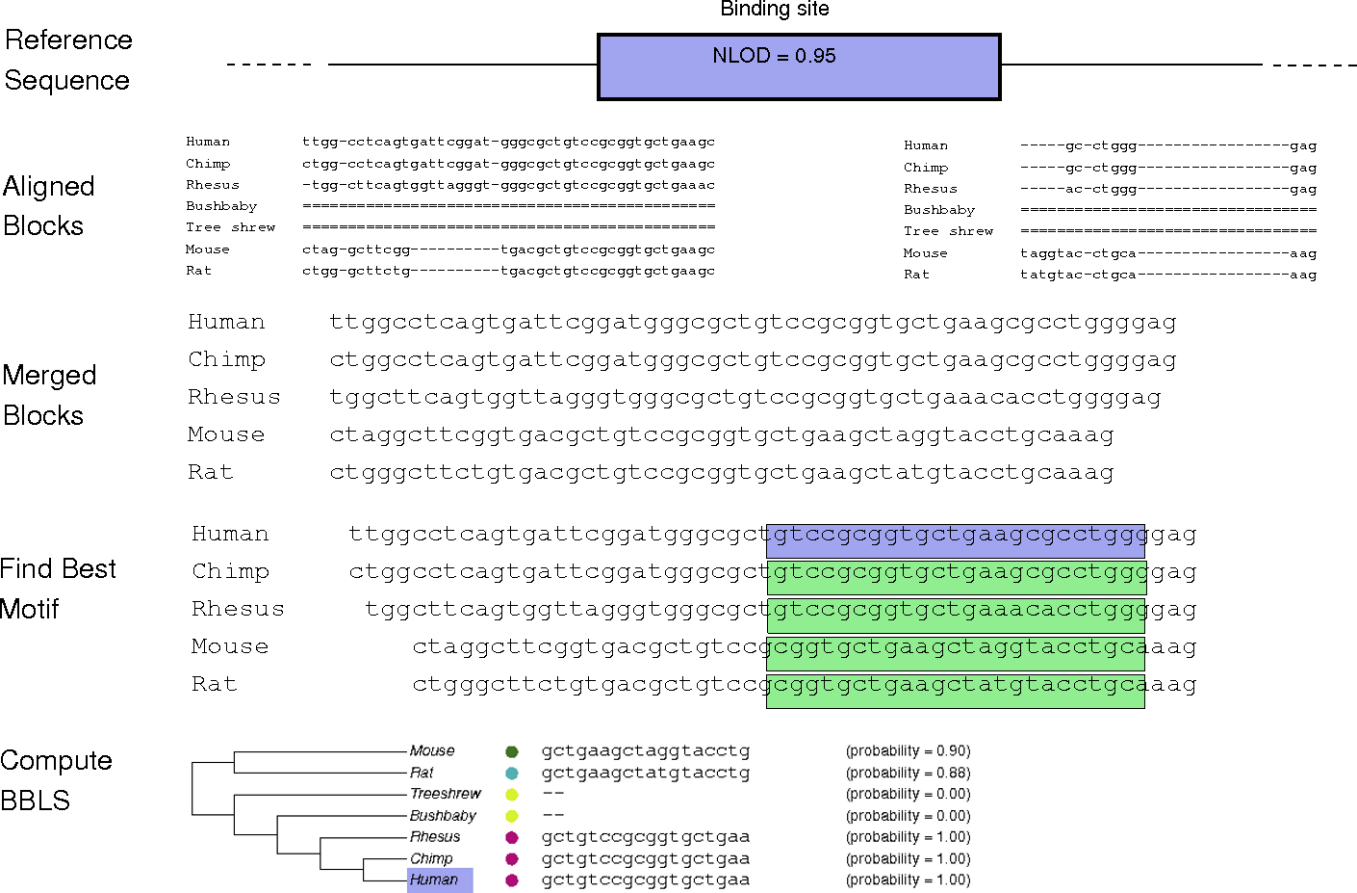
**Explanation of methods**. Diagram of updated methods. The reference genome is searched to find candidate sites and compute NLOD scores. Using the sequence around each site, overlapping aligned blocks of sequence are extracted from the multiple alignment. Nearby blocks are merged and then the best motif binding site in each species is found. The scores of the best motif sequences for each species are used to compute the BBLS score.

The prototype version of MotifMap searched the low-resolution multiple alignment files obtained from the UCSC Genome Browser [15] directly. As a result, possible alignments of a motif could be missed in other species, for example in poorly aligned regions with many gaps. To address this problem, the overall methodology used to search for aligned transcription factor binding sites has been considerably improved (Figure 1).

The new approach searches instead the reference genome directly and uses the low-resolution alignments only as a seed to identify regions in other species aligning to the motif in the reference species. An expanded sequence including 15 base pairs on each side of each binding site in the reference species is used to identify aligned regions in the other genomes. This expanded sequence helps compensate for the low-resolution nature of the whole genome alignments [16]. Furthermore, instead of using the aligned regions directly, which may be too short or contain many gaps, we find all the alignment blocks overlapping the expanded sequence. Due to the nature of the algorithm used to build the multiple alignments, the sequences in different aligning blocks for any single species may be very far apart from each other on the chromosome, or even on completely separate chromosomes. As a result, we only concatenate blocks that are within 30 base pairs of each other (maintaining any intervening sequence). This operation yields a set of blocks of aligning regions; each block contains sequences from other species aligned to the binding site. For each species, we find the motif sequence with the highest normalized log-odds score across all blocks. Finally, the scores corresponding to the selected sequence from each species are used for BBLS scoring. In practice, requiring a minimum number of species to be aligned to the reference sequence at each binding site improves performance. The default requirement, used for instance in the case of the yeast map, is set to at least one other species (i.e. BBLS *>*0). For the human map, in the public version of MotifMap, binding sites are required to be conserved across at least four non-primate species. This also enables a fair comparison to the prototype version that used the same requirement.

Because the new modular design of MotifMap is not dependent on searching the UCSC coarse multiple alignment files directly, it enables one to also use other alignments if necessary, such as high resolution alignments of the upstream regions of known homologous or orthologous genes, even when these are not in the UCSC format (e.g. the MAF format produced by the multiz alignment software), or to focus the search on any subset of the genome. To avoid bias from binding sites that occur in regions that are conserved for being part of a translated portion of a gene and are not necessarily under positive selection because of their importance for regulatory control, we exclude exonic regions of the genome from the default public version of MotifMap. Likewise, we exclude repetitive regions.

### Redundancy filter

A transcription factor is often annotated with multiple binding matrices in JASPAR and TRANSFAC. For example, each matrix may represent a specific isoform of the factor dependent on the biological context (e.g. cell type or experimental condition). However, in order to estimate a total number of unique potential binding sites, a given site can be counted only once for a given transcription factor, even when this factor has multiple binding matrices. For this purpose, we first perform the genome-wide search independently for each matrix, and then group overlapping binding sites. We choose a representative for each transcription factor in that group by picking the site with the highest BBLS score. The final result is a non-overlapping, non-redundant, list of binding sites for each transcription factor.

## Results

### New MotifMaps

Each MotifMap is generated automatically via a pipeline running on a parallel computer cluster. Comprehensive maps for human, mouse, fly, worm, and yeast have been generated and new maps can be produced automatically. Details about the genomes, alignments, and matrices used in each MotifMap can be seen in Table 1. The raw data for the total number of binding sites across the genomes ranges from hundreds of thousands for yeast, worm, and fly to millions for mouse and human. Table 2 summarizes the number of transcription factors, matrices, and binding sites for each available species after all filtering steps have been applied. For the human MotifMap, we predict 519,108 binding sites for 570 matrices, nearly a 5-fold increase over the number of sites and matrices in the prototype version, while maintaining a low FDR of 0.1 or less.

**Table 1:** Multiple alignment information

Species	Build	Alignment	# species in alignment	# of matrices
Yeast	sacCer2	multiz7way	7	507
Worm	ce6	multiz6way	6	6
Fly	dm3	multiz15way	12 (files only)^†^	262
Mouse	mm9	multiz30way	30	830
Human	hg18	multiz28way	17 (placentals only)^†^	837
Human	hg19	multiz46way	32 (placentals only)^†^	837

Number of species in multiple alignments for each reference species, and the number of original matrices for each species. Comprehensive maps exist for yeast, worm, fly, mouse, and human (both for a 17-species and 32-species alignment). See the UCSC Genome Browser website for more information on the alignments used and the species they contain. ^†^Trees used are truncated to remove the most distant species.

**Table 2:** Non-redundant transcription factor binding sites

Species	# Transcription Factors	# Matrices	# Sites	# Sites FDR ≤ 0.1
Yeast	161	147	115,387	1,577
Worm	6	6	88,895	69
Fly	94	66	191,655	36,091
Mouse	473	575	6,617,325	740,685
Human (hg18)	468	570	2,554,732	519,108
Human (hg19)	468	530	1,410,309	457,198

Number of non-redundant transcription factors and binding sites across the genome (see text for definition of "non-redundant").

### Evaluation of new methods using experimental data

We first compare the updated methodology to the prototype version using data on well-studied transcription factors and experimentally-determined binding sites using high-throughput methods, such as ChIP-seq. While ChIP-seq and related methods are not perfect, they still provide the best available experimental approximations to genome-wide maps of binding sites. While the prototype map used 17 species, a larger number of genomes and genome alignments has become available since its publication. Thus, for comparison purposes, we run the new methodology using both the same tree of 17 species used for the first prototype, as well as an expanded tree containing 32 placental mammals.

Specifically, we consider the same set of highly studied transcription factors (Table 3), same motifs, same experimental data [17-22], and same whole genome alignments as in Xie et al. [5], to compute the area under the Receiver Operating Characteristic (ROC) curves (AUC) using the updated methodology. For all motifs, we see an improvement of the AUC in the range of 1-5% over the previous version. [Note that when computing the AUC, we include all ChIP-seq regions that do not contain a conserved motif binding site in the class of true negatives, as in [5]. However, we still robustly observe improvements in the range of 0-5% when not including these regions in the class of true negatives.] For P53, CTCF, and NRSE, we observe an increase in the AUC with a decrease in the number of sites found. For NFKB and STAT1, we observe a modest increase in the number of sites along with an increase in the AUC. We also observe further modest improvements for a few of these transcription factors when the number of species in the multiple alignments is increased from 17 to 32 placental mammals (see the UCSC Genome Browser website for details on the species in each alignment).

**Table 3:** Performance comparison of the prototype and updated MotifMap pipelines

	NFKB	MYC	P53	STAT1	CTCF	NRSE
AUC						

Prototype, 17 species	0.722	0.683	0.861	0.606	0.814	0.941
Update, 17 species	**0.797**	0.765	**0.902**	0.780	0.887	0.950
Update, 32 species	0.786	**0.812**	0.896	**0.820**	**0.903**	**0.951**

Number of sites						

Prototype, 17 species	11,636	55,271	28,635	6,134	69,446	13,055
Update, 17 species	13,924	100,311	24,880	9,537	53,794	7,488
Update, 32 species	14,839	100,275	25,563	10,034	77,064	8,127

Area under the ROC curve (AUC) and number of sites found for the prototype MotifMap pipeline versus the updated MotifMap pipeline (performed with the original 17-species alignment and a newer 32-species alignment); the best performing method for each motif is shown in bold.

We also use ChIP-seq data available for 35 mouse transcription factors obtained from the TRANSFAC suite to further assess the performance of the MotifMap pipeline and compare it with other methods. We evaluate the performance of the BBLS scoring scheme to recover known binding sites identified by ChIP-seq against four other scores: BLS [13,14], NLOD (as described in this work), PhastCons [8], and PhyloP [9]. Each score is individually used to rank the binding sites identified by MotifMap. We calculate the number of true and false positive sites identified in the ChIP-seq data to compute the AUC, as in Xie et. al. [5]. Table 4 summarizes the results for the performance of the MotifMap pipeline in recovering the sites identified by the ChIP-seq methods by reporting the results for the 20 top transcription factors with the largest AUC values. For these 20 transcription factors, we see performances comparable to those seen for the human MotifMap: MotifMap achieves the best AUC result in 16 of them, while relatively small differences (3% or less) are seen for the remaining four, providing further evidence of the overall quality of the MotifMap system and its ability to generalize and identify binding sites in other species.

**Table 4:** Performance of the mouse MotifMap

Name	BBLS	BLS	NLOD	PhastCons	PhyloP
Ctcf	**0.901**	0.838	0.893	0.798	0.754
Myc:Max	**0.831**	0.826	0.731	0.773	0.690
Zfp281	**0.827**	0.820	0.611	0.691	0.679
Tcfcp211	**0.809**	0.500	0.754	0.800	0.668
c-Myc	**0.778**	0.771	0.734	0.758	0.710
Gli3	0.772	0.771	0.619	**0.806**	0.659
Gli1	**0.770**	0.728	0.727	0.704	0.689
E2f5	**0.760**	0.737	0.632	0.737	0.667
Myc	**0.760**	0.699	0.540	0.703	0.718
Pdx1	0.757	**0.765**	0.500	0.696	0.689
Trim28	**0.753**	0.749	0.609	0.640	0.642
Klf4	**0.740**	0.500	0.500	0.695	0.678
Esrrb	**0.739**	0.500	0.516	0.667	0.608
Zfa	**0.733**	0.731	0.660	0.677	0.644
Mycn	**0.730**	0.728	0.620	0.690	0.664
Cnot3	0.683	**0.688**	0.568	0.614	0.597
Stat3	**0.677**	0.634	0.656	0.655	0.614
Ppara	**0.673**	0.664	0.642	0.636	0.615
Nr0b1	**0.668**	0.653	0.598	0.612	0.597
Zfp42	0.629	0.627	0.596	0.650	**0.661**

Area under the ROC curve (AUC) for predicting transcription factor binding sites identified by ChIP-seq experiments in mouse. Each column is associated with a different method for scoring and ranking the putative sites identified by MotifMap, from which ROC curves and AUCs are computed. The best performing method for each motif is shown in bold.

### Localization analysis: binding site location properties

**Figure 2:**
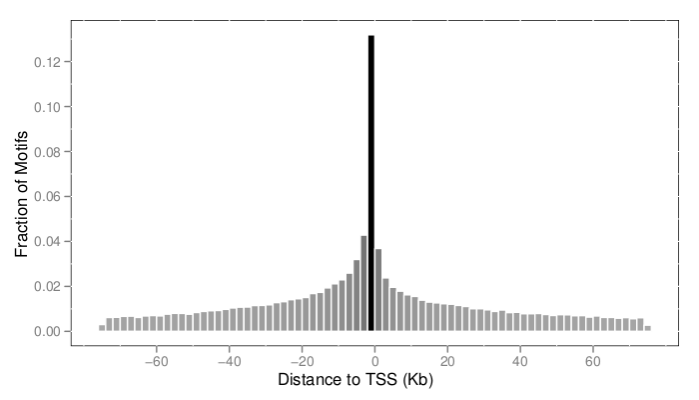
**Distribution of distance to closest gene for human binding sites**. Distribution of the distance to the closest gene (Transcription Start Site or TSS) for high confidence human motif binding sites.

**Figure 3:**
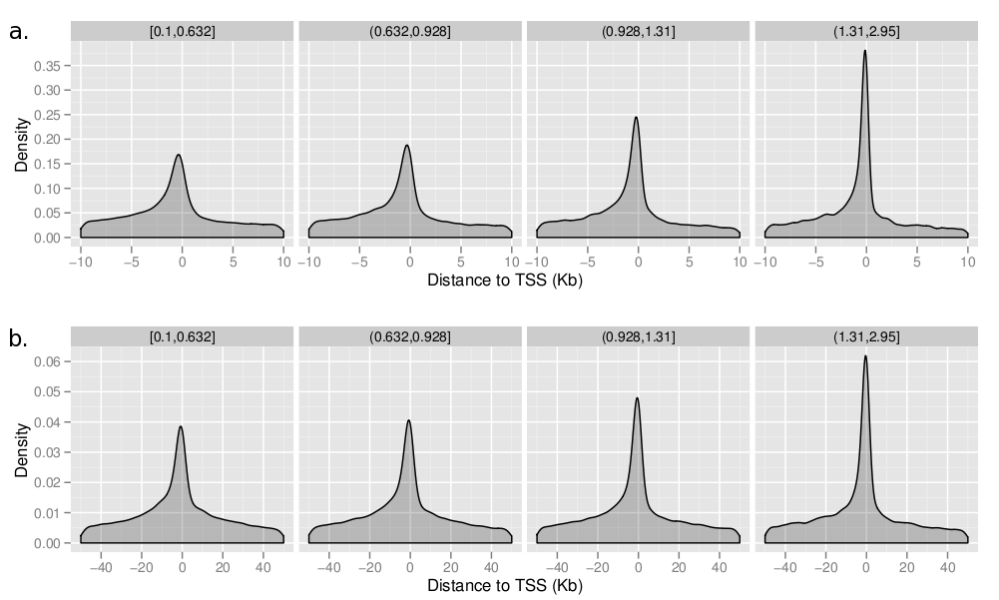
**Distribution of MotifMap regulatory elements as a function of conservation**. Empirical distribution of distances of human transcription factor binding sites to the closest (≤ 10 Kbp and ≤ 50 Kbp) RefSeq gene transcription start site (TSS). The sites are grouped into quartiles according to the BBLS score; each group has one quarter of the total binding sites. The BBLS range for each quartile is given at the top of each plot. As the BBLS conservation score increases, we observe a larger proportion of binding sites close to the TSS of the closest gene.

To further assess the quality of the maps, we examine the distribution of the candidate sites relative to the locations of genes across the genome. Using the high confidence data (FDR ≤ 0.1), we find that the majority of sites are within 1 Kbp of the transcription start sites (TSS) of known genes across all species. Figure 2 shows a plot of the distribution of distance to the closest gene for each binding site for the human genome. This distribution becomes increasingly peaked as one increases the BBLS threshold filter (Figures 3a, b). However, we note that we also find high-confidence sites significantly far from known transcription start sites (further than 100 Kbp away). These sites would be missed in a promoter-only analysis of transcription factor binding sites. We see similar distributions for mouse, while for smaller genomes (such as yeast and fly) the binding sites are even closer to the transcription start sites. This is expected, since the genomes of these species are more condensed, including shorter promoter and intragenic regions.

### MotifMap system, web server, and data integration

The MotifMap "system" consists of three main components: (1) a computational pipeline to perform the genome-wide search; (2) a database to store candidate motif binding sites, the scores associated with them, and the relationships to other features; (3) custom code to interface between the database and a web service; and (4) a Flex web application, to display data to users. All steps in the pipeline for identifying and scoring binding sites are performed in parallel using a high performance computer cluster. Along with the locations and scores for each binding site, we compile and store relationships between the binding sites and other genomic features, such as genes (RefSeq [23] and Ensembl [24]) and Gene Ontology (GO) annotations [25]. Some species (fly and yeast) use specific gene annotation resources instead (FlyBase [26] and SGD [27]). The database is currently being expanded as other MotifMaps and new relationships become available. The binding site data and relationships for all available species are publicly available through the MotifMap web site (http://motifmap.igb.uci.edu).

While the prototype MotifMap version had a simple interface to display data, the new web application has been extensively upgraded with multiple features and functionalities to allow users to explore these genome-wide datasets more easily. User can interactively select a model species and one or more transcription factors, visualize the logos of the corresponding motifs, filter the results by various criteria and thresholds (genome location, NLOD/z-score, BBLS, FDR), and retrieve a corresponding list of binding sites, with the distances to the nearest TSS and the corresponding gene annotations. The results can be downloaded in a variety of standard formats (GFF, BED, CSV) or exported directly for visualization in the UCSC Genome Browser. Furthermore, for each motif binding site, users can view the local multiple alignment and the phylogenetic tree with the corresponding probability scores for each species, as shown in simplified form at the bottom of Figure 1. A Python implementation of an efficient algorithm for computing the Bayesian Branch Length Score can also be downloaded from the MotifMap web site. MotifMap uses an integrative approach combining, for instance, phylogenetic, genomic, and transcription factor data. The resulting maps themselves can in turn be integrated with many other datasets (see Discussion). Two kinds of data that are fully integrated into the MotifMap database and available to the user are GO annotations and SNPs. For instance, for a given GO annotation and the corresponding set of genes, user can retrieve all the nearby candidate binding sites. Likewise, SNPs falling within or near a transcription factor binding site have the potential for influencing the regulation of the corresponding gene [28]. Thus it is useful to be able to list which SNPs in a GWAS (Genome Wide Association Study) or other genotyping study fall within or nearby transcription factor binding sites. Analyses of GWAS data focused primarily on coding regions run the risk of missing important SNPs affecting regulatory regions. The relationship between SNPs and binding sites has been integrated into the MotifMap web application as an additional analysis tool called SNPer, which allows the retrieval of motif binding sites that overlap with SNP sites. The HapMap3 [29] and dbSNP [30] datasets are currently available for use with the mouse and human MotifMap. Users can download the MotifMap results for further integration with specific GWAS or other studies.

## Discussion

The MotifMap approach has allowed us to derive state-of-the-art genome-wide maps of candidate regulatory elements for some of the main model organisms, in particular for mouse and human. For the worm, the map produced is considerably more primitive because only six transcription factor binding matrices are available in TRANSFAC and JASPAR. However, the availability of the map for this limited set of transcription factors may still be of some use and all the maps will be updated as more binding matrices become available.

Each binding site predicted by MotifMap corresponds in fact to a regulatory hypothesis, thus a single MotifMap can generate from thousands to millions of hypotheses. These hypotheses can be tested and refined in the laboratory, either individually in the case of very specific interactions which can be tested with great precision, or on a larger but less precise scale using high-throughput methods, such as ChIP-seq. These multiple hypotheses can also be further refined and analyzed by computational methods using integrative approaches where regulatory hypotheses are simultaneously combined: (1) with each other in the form of regulatory networks; and (2) with other kinds of data. Regulatory hypotheses can be integrated with each other to identify regulatory networks of transcription factors, including regulatory loops and, for instance, hypothesize that transcription factor A regulates transcription factor B, transcription factor B regulates transcription factor C, and transcription factor C regulates transcription factor A. These networks and loops can be thought of as the core regulatory network of a cell. Regulatory hypotheses can also be integrated with many other kinds of data to refine regulatory inferences, as described in the Results section using GO and SNP data and below with other kinds of data. In particular, MotifMap and GO annotations can be used to infer the common functions of a set of genes targeted by a transcription factor or, conversely, to infer the transcription factor that may regulate a set of genes with common GO annotations. To illustrate these ideas, here we give a simple demonstration of the power of integrating MotifMap and other data to generate regulatory network hypotheses, above the level of an individual regulatory site. For demonstration purposes, we choose two examples. We reconstruct the P53 apoptotic pathway, since it is an important and well-studied pathway which allows us to assess the quality of the predictions. We also apply the same general ideas to the Gli family of transcription factors and the hedgehog pathway to demonstrate the effectiveness of these methods on a relatively less-studied transcription factor family and pathway where important regulatory effects remain to be discovered.

### Mouse P53 apoptotic pathway

**Figure 4:**
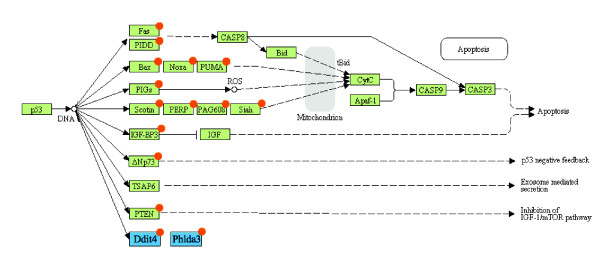
**Known apoptotic targets of P53**. Known apoptotic genes from the KEGG pathway database and the literature for P53. Genes in light green are annotated in KEGG. Orange dots indicate direct targets recovered by MotifMap. DDIT4 and PHLDA3 are examples of additional direct targets identified by MotifMap with FDR < 0.05 which have been reported in the literature but are not present in KEGG.

We attempt to reconstruct the P53 direct regulatory interactions in the mouse P53 apoptotic pathway using data from MotifMap for putative P53 binding sites across the genome. We first compile a list of over 380 unique gene transcripts from the RefSeq database [23] annotated with the Gene Ontology term "Apoptosis" (GO:0006915). We then retrieve predicted P53 binding sites from MotifMap in the promoter region of these genes to generate a regulatory network of P53's role in apoptosis. The promoter region of a gene is defined as 15 Kbp upstream and 3 Kbp downstream, which approximately encompasses the region associated with the first intron, from the transcription start site. To evaluate the network generated from MotifMap data, we compare it to the P53 pathway described in the KEGG database [31], which reports 14 genes directly regulated by P53 in the apoptotic pathway (Figure 4). Table 5 shows the number of known and potentially novel P53 targets predicted as a function of FDR. At a FDR of 0.05, we predict eight target genes from the list of all apoptotic genes, six of which are annotated in KEGG. Searching the literature reveals that the other two target proteins, DDIT4 and PHLDA3, are also known targets of P53 [32,33] but not annotated in KEGG. At a FDR of 0.25, we predict a total of 71 targets, including 12 of the 14 targets annotated in KEGG; the only exceptions are FAS and TSAP6 (also called STEAP3). FAS is a predicted direct target, but has a slightly higher FDR (0.28). For TSAP6 we find two P53 sites (1784 bp and 4582 bp upstream) with a strong motif matching score; however these sites are not conserved. A novel predicted target is BID, which is annotated in KEGG as a downstream indirect target in the P53 apoptotic pathway. If we reduce the length of the upstream promoter regions from 15 Kbp down to 5 Kbp, the same KEGG targets are recovered with the exception of PIDD and SHSA5. A few targets have P53 binding sites downstream of the TSS, in the first intron, and these would not have been recovered with a search focused on promoter regions only. Thus in short the MotifMap system is capable of robustly recovering most of the direct targets of P53 described in KEGG, as well as providing a ranked list of potential new targets, some of which can be confirmed by a literature search.

**Table 5:** Mouse P53 apoptotic pathway

FDR	0	0.05	0.1	0.15	0.2	0.25
KEGG known	4	6	6	7	10	12
Potentially novel	1	2	16	29	50	73

Total	5	8	22	36	60	85

Number of known (annotated in KEGG) and potentially novel P53 direct targets predicted at different FDR thresholds.

### Mouse Gli hedgehog pathway

**Figure 5:**
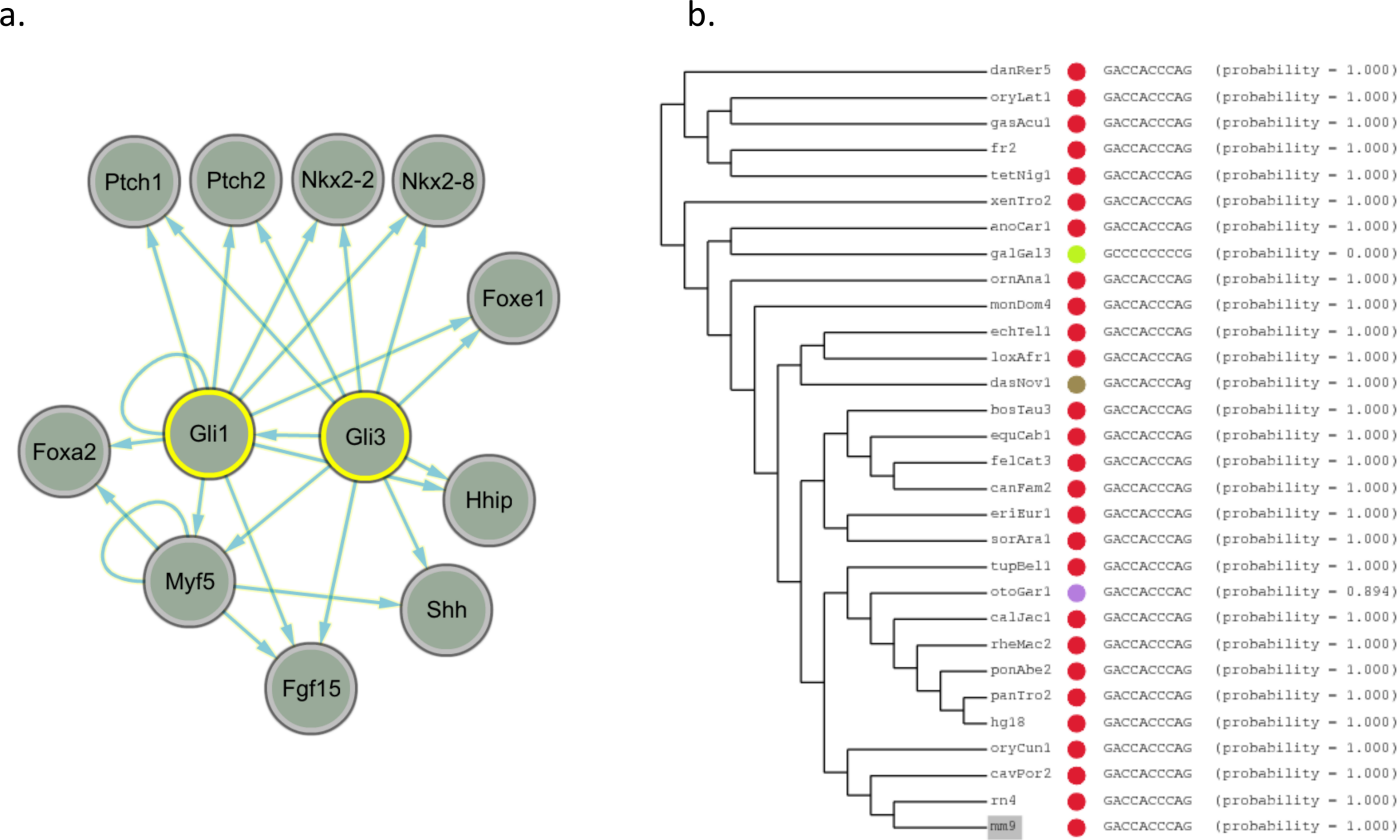
**Targets of Gli in the hedgehog pathway and motif alignment of a highly conserved Gli1 binding site**. Network showing the known Gli targets in mouse. All direct targets were recovered by MotifMap, including the autoregulatory loop of Gli1. Nkx2-2, Nkx2-8 and Ptch2 are examples of additional direct targets identified by MotifMap with binding site conserved in more than 25 out of the 30 species in the genome alignment. **(5b) Motif alignment for a highly conserved Gli1 binding site** Motif alignment for a highly conserved Gli1 binding site 1365 bp upstream of the Nkx2-8 transcription start site is also shown.

Next, we examine the Gli family of transcription factors. Although Gli is a relatively less studied transcription factor, mutations in Gli genes have been associated with multiple developmental disorders and cancers [34]. We first compile a list of Gli targets. The KEGG database lists only two annotated targets of Gli1 (Hhip and Ptch1), as well as an autoregulatory loop of Gli1. Gli1 is annotated as a downstream effector of the Sonic hedgehog pathway [34]. In addition, Gli1 is known to regulate the Wnt signaling pathways [35]. Due to the lack of many annotated targets in KEGG, we used the Transcriptional Regulatory Element Database (TRED) [36], which contains an additional four annotated Gli family targets. We find Gli binding sites predicted by MotifMap in the promoter region of the seven annotated targets and also many of the Wnt proteins. We observe predicted binding sites in the Shh promoter (14,843 bp upstream) as well as in the second intron. In addition, we recover the Gli1 autoregulatory loop [37] and regulation of Gli3 by Gli1 [38] (Figure 5a). All binding sites for all targets are recovered at an estimated FDR ≤ 0.25, within 15 Kbp upstream and 3 Kbp downstream of each gene. Furthermore, we identify a highly conserved binding site (BBLS *>*7, perfectly conserved in 27 out of the 30 species in the alignment) near Ptch1. Nkx2-8 and Nkx2-2, both of which have been reported as targets of Gli family transcription factors [39,40], have predicted binding sites within 2 Kbp upstream of the transcription start site with similar conservation (Figure 5b). We also identify Rab34 as a true Gli target [39] at a lower conservation level (BBLS *>*2); this threshold includes approximately 100 novel targets.

### Further integration and challenges

Regulatory networks do not consist only of transcription factors and their direct regulatory interactions, but can include also protein-protein interactions (PPI). Integrating PPI (physical or genetic) data [41,42] with protein-DNA interactions from MotifMap can yield a more comprehensive view of molecular mechanisms and networks. Integration of PPI data can also facilitate the identification of transcriptional complexes. For example, evidence for a complex based on adjacency of binding sites for two transcription factors could be strengthened by data supporting physical interactions between these factors. In general, however, factors with proximal binding sites need not physically interact with each other in order to influence transcription, and MotifMap data can be used to identify modules of transcription factors with co-occurring binding sites near co-regulated genes. To derive a more accurate and complete global picture, it is also important to incorporate information about RNA elements involved in gene regulation [43]. As so far described, MotifMap provides a static view of potential transcription-factor/DNA interactions. Since transcription factor regulation of most genes does not occur ubiquitously or constantly across all cells in an organism, DNA microarrays and high-throughput sequencing of transcripts (RNA-seq) provide another important source of information about the cell-specific, tissue-specific, or condition-specific expression of genes. Thus MotifMap can be integrated with gene expression data, such as the Gene Expression Omnibus (GEO) data [44]. This integration provides additional information about, for instance, the average direction of a particular interaction (up- or down-regulation) across many experiments, or about the specific portion of the total potential regulatory network that is activated in a given condition. An important challenge ahead lies in better understanding the role of epigenetics in the regulation of gene transcription. An interesting source of data for further integration with MotifMap comes from the ENCODE project [45] providing the locations of epigenetic signatures, such as histone tail methylations or acetylations, across the human genome for a large number of cell lines. Combinations of these markers can identify transcription factor binding sites that are specific to a particular cell line; for example, the presence of H3K4Me1 and absence of H3K4Me3 denotes enhancer regions. This integration induces regulatory sub-networks, potentially describing important interactions needed for a particular cell type to function properly.

Another considerable challenge is the role of chromatin and 3D structure in gene regulation. New high-throughput techniques like Chromosome Conformation Capture-on-Chip (4C), Hi-C and Chromatin Interaction Analysis using Paired-End Tag sequencing (ChIA-PET) allow the detection of long range or inter-chromosomal interactions of DNA [46-48]. This provides the ability to detect regulatory elements that may be distal to the gene they regulate linearly, but are brought close together in 3-dimensional space. For instance, a recent study used 4C to investigate the properties and dynamics of the genomic loci that are in contact with glucocorticoid receptor (GR) responsive loci [49]. Incorporating this kind of data into MotifMap could provide further evidence of these distant regulatory interactions and improve our ability to infer regulatory mechanisms and networks.

Many other data, such as scientific literature, or information about diseases and drugs, are also being integrated in house with MotifMap. Each data comes with its own noise and limitations and it is the combination of diverse lines of evidence that has the power to solidify inferences and rank hypotheses in a relevant way. This integration process is not new, of course, and in essence is at the root of IBM's Watson system for the game of Jeopardy [50]. This integration process is ongoing and raises computational challenges both in its execution and in what can be served publicly given a limited amount of computational resources.

Finally, another potential computational challenge for systems like MotifMap is the dynamic use of evolutionary trees and comparative genomics. The current version of MotifMap builds a genome-wide map, assessing conservation with a single static tree for each species. But clearly not all regulatory elements are conserved, and even when they are, the optimal tree for assessing their degree of conservation may vary with each transcription factor and each biological question. Thus studying how to dynamically assess conservation, including its weaker forms [51,52], and how to discover regulatory elements that are poorly conserved remain important questions for further investigations.

## Conclusion

The MotifMap system aims to provide comprehensive genome-wide map of regulatory elements for each organism. Since experimental data on gene expression obtained with DNA microarray or high-throughput sequencing methods is inherently biased (to a specific condition, cell type, etc.), a resource that catalogs transcription factor binding sites across the entire genome in an unbiased fashion is valuable. We have created the first such comprehensive maps of candidate regulatory motifs across the yeast, fly, worm, mouse, and human genomes. The updated methodology has improved the detection of experimentally validated motif binding sites and, together with integration with other data, the generation of regulatory networks and hypotheses. Overlaying and integrating information from multiple sources, well beyond transcription factor binding motifs and genomic DNA sequences, is key to building better maps and ultimately to understanding gene regulation on a genome-wide scale.

## Authors' contributions

PB conceived the study and the algorithms and coordinated and supervised all aspects. XX contributed to the algorithms and the coordination. KD, VP, and PB wrote the manuscript. PR, VP, and KD wrote the software and implemented the web server. KD, VP, and PB performed the detailed analyses. All authors proofread and approved the final manuscript.

## Supplementary Material

Additional file 1 **Sources of binding matrices**. Table listing the original source of each transcription factor binding matrix. Click here for file
