# Supplementary material for: MotifMap: integrative genome-wide maps of regulatory motif sites for model species
Source: BMC Bioinformatics. 2011 Dec 30;12:495. doi: 10.1186/1471-2105-12-495 (PMC3293935; doi:10.1186/1471-2105-12-495)
Supplement: Additional file 1 — Sources of binding matrices. Table listing the original source of each transcription factor binding matrix. [file 1471-2105-12-495-S1.PDF]

1. Sources of transcription factor binding matrices.

Species	Citations
Yeast	[1–5]
Worm	[4, 5]
Fly	[6–56]
Mouse	[4, 5]
Human	[4, 5]

References

[1] MacIsaac K, Wang T, Gordon DB, Gifford D, Stormo G, Fraenkel E: **An improved map of conserved regulatory sites for Saccharomyces cerevisiae**. *BMC Bioinformatics* 2006, **7**:113+, [[http://dx.doi.org/10.1186/1471-2105-7-113]].

[2] Harbison CT, Gordon DB, Lee TI, Rinaldi NJ, Macisaac KD, Danford TW, Hannett NM, Tagne JB, Reynolds DB, Yoo J, Jennings EG, Zeitlinger J, Pokholok DK, Kellis M, Rolfe PA, Takusagawa KT, Lander ES, Gifford DK, Fraenkel E, Young RA: **Transcriptional regulatory code of a eukaryotic genome**. *Nature* 2004, **431**(7004):99–104, [[http://dx.doi.org/10.1038/nature02800]].

[3] Lee TI, Rinaldi NJ, Robert F, Odom DT, Bar-Joseph Z, Gerber GK, Hannett NM, Harbison CT, Thompson CM, Simon I, Zeitlinger J, Jennings EG, Murray HL, Gordon DB, Ren B, Wyrick JJ, Tagne JB, Volkert TL, Fraenkel E, Gifford DK, Young RA: **Transcriptional Regulatory Networks in Saccharomyces cerevisiae**. *Science* 2002, **298**(5594):799–804, [[http://dx.doi.org/10.1126/science.1075090]].

[4] Matys V, Fricke E, Geffers R, Gössling E, Haubrock M, Hehl R, Hornischer K, Karas D, Kel AE, Kel-Margoulis OV, Kloos DUU, Land S, Lewicki-Potapov B, Michael H, Münch R, Reuter I, Rotert S, Saxel H, Scheer M, Thiele S, Wingender E: **TRANSFAC: transcriptional regulation, from patterns to profiles.** *Nucleic acids research* 2003, **31**:374–378, [[http://dx.doi.org/10.1093/nar/gkg108]].

[5] Portales-Casamar E, Thongjuea S, Kwon AT, Arenillas D, Zhao X, Valen E, Yusuf D, Lenhard B, Wasserman WW, Sandelin A: **JASPAR 2010: the greatly expanded open-access database of transcription factor binding profiles.** *Nucleic acids research* 2010, **38**(Database issue):D105–110, [[http://dx.doi.org/10.1093/nar/gkp950]].

[6] Nellesen DT, Lai EC, Posakony JW: **Discrete enhancer elements mediate selective responsiveness of enhancer of split complex genes to common transcriptional activators.** *Developmental biology* 1999, **213**:33–53, [[http://www.ncbi.nlm.nih.gov/pubmed/10452845]].

[7] Kremser T, Gajewski K, Schulz RA, Renkawitz-Pohl R: **Tinman regulates the transcription of the beta3 tubulin gene (betaTub60D) in the dorsal vessel of Drosophila.** *Developmental biology* 1999, **216**:327–339, [[http://www.ncbi.nlm.nih.gov/pubmed/10588882]].

[8] Lee S, Garfinkel MD: **Characterization of Drosophila OVO protein DNA binding specificity using random DNA oligomer selection suggests zinc finger degeneration.** *Nucleic acids research* 2000, **28**(3):826–834, [[http://www.ncbi.nlm.nih.gov/pubmed/10637336]].

[9] Shimell MJ, Peterson AJ, Burr J, Simon JA, O'Connor MB: **Functional analysis of repressor binding sites in the iab-2 regulatory region of the abdominal-A homeotic gene.** *Developmental biology* 2000, **218**:38–52, [[http://www.ncbi.nlm.nih.gov/pubmed/10644409]].

[10] Dai H, Hogan C, Gopalakrishnan B, Torres-Vazquez J, Nguyen M, Park S, Raftery LA, Warrior R, Arora K: **The zinc finger protein schnurri acts as a Smad partner in mediating the transcriptional response to decapentaplegic.** *Developmental biology* 2000, **227**(2):373–387, [[http://www.ncbi.nlm.nih.gov/pubmed/11071761]].

[11] Zhang H, Levine M, Ashe HL: **Brinker is a sequence-specific transcriptional repressor in the Drosophila embryo.** *Genes & development* 2001, **15**(3):261–266, [[http://www.ncbi.nlm.nih.gov/pubmed/11159907]].

[12] Takata K, Yoshida H, Hirose F, Yamaguchi M, Kai M, Oshige M, Sakimoto I, Koiwai O, Sakaguchi K: **Drosophila mitochondrial transcription factor A:**

characterization of its cDNA and expression pattern during development. *Biochemical and biophysical research communications* 2001, **287**(2):474–483, [[http://www.ncbi.nlm.nih.gov/pubmed/11554753]].

[13] Zaffran S, Küchler A, Lee HH, Frasch M: **biniou (FoxF), a central component in a regulatory network controlling visceral mesoderm development and midgut morphogenesis in Drosophila.** *Genes & development* 2001, **15**(21):2900–2915, [[http://www.ncbi.nlm.nih.gov/pubmed/11691840]].

[14] Kusch T, Storck T, Walldorf U, Reuter R: **Brachyury proteins regulate target genes through modular binding sites in a cooperative fashion.** *Genes & development* 2002, **16**(4):518–529, [[http://www.ncbi.nlm.nih.gov/pubmed/11850413]].

[15] Wang LH, Chmelik R, Nirenberg M: **Sequence-specific DNA binding by the vnd/NK-2 homeodomain of Drosophila.** *Proceedings of the National Academy of Sciences of the United States of America* 2002, **99**(20):12721–12726, [[http://www.ncbi.nlm.nih.gov/pubmed/12232052]].

[16] Bhaskar V, Courey AJ: **The MADF-BESS domain factor Dip3 potentiates synergistic activation by Dorsal and Twist.** *Gene* 2002, **299**(1-2):173–184, [[http://www.ncbi.nlm.nih.gov/pubmed/12459265]].

[17] Lunde K, Trimble JL, Guichard A, Guss KA, Nauber U, Bier E: **Activation of the knirps locus links patterning to morphogenesis of the second wing vein in Drosophila.** *Development (Cambridge, England)* 2003, **130**(2):235–248, [[http://www.ncbi.nlm.nih.gov/pubmed/12466192]].

[18] Christianson AM, King DL, Hatzivassiliou E, Casas JE, Hallenbeck PL, Nikodem VM, Mitsialis SA, Kafatos FC: **DNA binding and heteromerization of the Drosophila transcription factor chorion factor 1/ultraspiracle.** *Proceedings of the National Academy of Sciences of the United States of America* 1992, **89**(23):11503–11507, [[http://www.ncbi.nlm.nih.gov/pubmed/1280827]].

[19] Gogos JA, Hsu T, Bolton J, Kafatos FC: **Sequence discrimination by alternatively spliced isoforms of a DNA binding zinc finger domain.** *Science (New York, N.Y.)* 1992, **257**(5078):1951–1955, [[http://www.ncbi.nlm.nih.gov/pubmed/1290524]].

[20] Lours C, Bardot O, Godt D, Laski FA, Couderc JL: **The Drosophila melanogaster BTB proteins bric à brac bind DNA through a composite DNA binding domain containing a pipsqueak and an AT-Hook motif.** *Nucleic acids research* 2003, **31**(18):5389–5398, [[http://www.ncbi.nlm.nih.gov/pubmed/12954775]].

[21] Hoch M, Gerwin N, Taubert H, Jäckle H: **Competition for overlapping sites in the regulatory region of the Drosophila gene Krüppel.** *Science (New York, N.Y.)* 1992, **256**(5053):94–97, [[http://www.ncbi.nlm.nih.gov/pubmed/1348871]].

[22] Yan SJ, Gu Y, Li WX, Fleming RJ: **Multiple signaling pathways and a selector protein sequentially regulate Drosophila wing development.** *Development (Cambridge, England)* 2004, **131**(2):285–298, [[http://www.ncbi.nlm.nih.gov/pubmed/14701680]].

[23] Senger K, Armstrong GW, Rowell WJ, Kwan JM, Markstein M, Levine M: **Immunity regulatory DNAs share common organizational features in Drosophila.** *Molecular cell* 2004, **13**:19–32, [[http://www.ncbi.nlm.nih.gov/pubmed/14731391]].

[24] Pan D, Courey AJ: **The same dorsal binding site mediates both activation and repression in a context-dependent manner.** *The EMBO journal* 1992, **11**(5):1837–1842, [[http://www.ncbi.nlm.nih.gov/pubmed/1582412]].

[25] Bilioni A, Craig G, Hill C, McNeill H: **Iroquois transcription factors recognize a unique motif to mediate transcriptional repression in vivo.** *Proceedings of the National Academy of Sciences of the United States of America* 2005, **102**(41):14671–14676, [[http://www.ncbi.nlm.nih.gov/pubmed/16203991]].

[26] Ekker SC, Young KE, von Kessler DP, Beachy PA: **Optimal DNA sequence recognition by the Ultrabithorax homeodomain of Drosophila.** *The EMBO journal* 1991, **10**(5):1179–1186, [[http://www.ncbi.nlm.nih.gov/pubmed/1673656]].

[27] Urness LD, Thummel CS: **Molecular interactions within the ecdysone regulatory hierarchy: DNA binding properties of the Drosophila ecdysone-inducible E74A protein.** *Cell* 1990, **63**:47–61, [[http://www.ncbi.nlm.nih.gov/pubmed/2208281]].

[28] England BP, Heberlein U, Tjian R: **Purified Drosophila transcription factor, Adh distal factor-1 (Adf-1), binds to sites in several Drosophila promoters and activates transcription.** *The Journal of biological chemistry* 1990, **265**(9):5086–5094, [[http://www.ncbi.nlm.nih.gov/pubmed/2318884]].

[29] Stanojević D, Hoey T, Levine M: **Sequence-specific DNA-binding activities of the gap proteins encoded by hunchback and Krüppel in Drosophila.** *Nature* 1989, **341**(6240):331–335, [[http://www.ncbi.nlm.nih.gov/pubmed/2507923]].

[30] Dearolf CR, Topol J, Parker CS: **The caudal gene product is a direct activator of fushi tarazu transcription during Drosophila embryogenesis.** *Nature* 1989, **341**(6240):340–343, [[http://www.ncbi.nlm.nih.gov/pubmed/2571934]].

[31] Dynlacht BD, Attardi LD, Admon A, Freeman M, Tjian R: **Functional analysis of NTF-1, a developmentally regulated Drosophila transcription factor that binds neuronal cis elements.** *Genes & development* 1989, **3**(11):1677–1688, [[http://www.ncbi.nlm.nih.gov/pubmed/2606344]].

[32] Hoey T, Levine M: **Divergent homeo box proteins recognize similar DNA sequences in Drosophila.** *Nature* 1988, **332**(6167):858–861, [[http://www.ncbi.nlm.nih.gov/pubmed/2895896]].

[33] Häcker U, Kaufmann E, Hartmann C, Jürgens G, Knöchel W, Jäckle H: **The Drosophila fork head domain protein crocodile is required for the establishment of head structures.** *The EMBO journal* 1995, **14**(21):5306–5317, [[http://www.ncbi.nlm.nih.gov/pubmed/7489720]].

[34] Murphy AM, Lee T, Andrews CM, Shilo BZ, Montell DJ: **The breathless FGF receptor homolog, a downstream target of Drosophila C/EBP in the developmental control of cell migration.** *Development (Cambridge, England)* 1995, **121**(8):2255–2263, [[http://www.ncbi.nlm.nih.gov/pubmed/7671793]].

[35] Wilson D, Sheng G, Lecuit T, Dostatni N, Desplan C: **Cooperative dimerization of paired class homeo domains on DNA.** *Genes & development* 1993, **7**(11):2120–2134, [[http://www.ncbi.nlm.nih.gov/pubmed/7901121]].

[36] Ekker SC, Jackson DG, von Kessler DP, Sun BI, Young KE, Beachy PA: **The degree of variation in DNA sequence recognition among four Drosophila homeotic proteins.** *The EMBO journal* 1994, **13**(15):3551–3560, [[http://www.ncbi.nlm.nih.gov/pubmed/7914870]].

[37] Fuse N, Hirose S, Hayashi S: **Diploidy of Drosophila imaginal cells is maintained by a transcriptional repressor encoded by escargot.** *Genes & development* 1994, **8**(19):2270–2281, [[http://www.ncbi.nlm.nih.gov/pubmed/7958894]].

[38] Van Doren M, Bailey AM, Esnayra J, Ede K, Posakony JW: **Negative regulation of proneural gene activity: hairy is a direct transcriptional repressor of achaete.** *Genes & development* 1994, **8**(22):2729–2742, [[http://www.ncbi.nlm.nih.gov/pubmed/7958929]].

[39] Ades SE, Sauer RT: **Differential DNA-binding specificity of the engrailed homeodomain: the role of residue 50.** *Biochemistry* 1994, **33**(31):9187–9194, [[http://www.ncbi.nlm.nih.gov/pubmed/8049221]].

[40] von Kalm L, Crossgrove K, Von Seggern D, Guild GM, Beckendorf SK: **The Broad-Complex directly controls a tissue-specific response to the steroid hormone ecdysone at the onset of Drosophila metamorphosis.** *The EMBO journal* 1994, **13**(15):3505–3516, [[http://www.ncbi.nlm.nih.gov/pubmed/8062827]].

[41] Hirose F, Yamaguchi M, Handa H, Inomata Y, Matsukage A: **Novel 8-base pair sequence (Drosophila DNA replication-related element) and specific binding factor involved in the expression of Drosophila genes for DNA polymerase alpha and proliferating cell nuclear antigen.** *The Journal of biological chemistry* 1993, **268**(3):2092–2099, [[http://www.ncbi.nlm.nih.gov/pubmed/8093616]].

[42] Mauhin V, Lutz Y, Dennefeld C, Alberga A: **Definition of the DNA-binding site repertoire for the Drosophila transcription factor SNAIL.** *Nucleic acids research* 1993, **21**(17):3951–3957, [[http://www.ncbi.nlm.nih.gov/pubmed/8371971]].

[43] Yan R, Small S, Desplan C, Dearolf CR, Darnell JE: **Identification of a Stat gene that functions in Drosophila development.** *Cell* 1996, **84**(3):421–430, [[http://www.ncbi.nlm.nih.gov/pubmed/8608596]].

[44] Gregory SL, Kortschak RD, Kalionis B, Saint R: **Characterization of the dead ringer gene identifies a novel, highly conserved family of sequence-specific DNA-binding proteins.** *Molecular and cellular biology* 1996, **16**(3):792–799, [[http://www.ncbi.nlm.nih.gov/pubmed/8622680]].

[45] Wilson DS, Sheng G, Jun S, Desplan C: **Conservation and diversification in homeodomain-DNA interactions: a comparative genetic analysis.** *Proceedings of the National Academy of Sciences of the United States of America* 1996, **93**(14):6886–6891, [[http://www.ncbi.nlm.nih.gov/pubmed/8692913]].

[46] Jun S, Desplan C: **Cooperative interactions between paired domain and homeodomain.** *Development (Cambridge, England)* 1996, **122**(9):2639–2650, [[http://www.ncbi.nlm.nih.gov/pubmed/8787739]].

[47] Akiyama Y, Hosoya T, Poole AM, Hotta Y: **The gcm-motif: a novel DNA-binding motif conserved in Drosophila and mammals.** *Proceedings of the National Academy of Sciences of the United States of America* 1996, **93**(25):14912–14916, [[http://www.ncbi.nlm.nih.gov/pubmed/8962155]].

[48] Erdman SE, Chen HJ, Burtis KC: **Functional and genetic characterization of the oligomerization and DNA binding properties of the Drosophila doublesex proteins.** *Genetics* 1996, **144**(4):1639–1652, [[http://www.ncbi.nlm.nih.gov/pubmed/8978051]].

[49] van de Wetering M, Cavallo R, Dooijes D, van Beest M, van Es J, Loureiro J, Ypma A, Hursh D, Jones T, Bejsovec A, Peifer M, Mortin M, Clevers H: **Armadillo coactivates transcription driven by the product of the Drosophila segment polarity gene dTCF.** *Cell* 1997, **88**(6):789–799, [[http://www.ncbi.nlm.nih.gov/pubmed/9118222]].

[50] Lam GT, Jiang C, Thummel CS: **Coordination of larval and prepupal gene expression by the DHR3 orphan receptor during Drosophila metamorphosis.** *Development (Cambridge, England)* 1997, **124**(9):1757–1769, [[http://www.ncbi.nlm.nih.gov/pubmed/9165123]].

[51] Haenlin M, Cubadda Y, Blondeau F, Heitzler P, Lutz Y, Simpson P, Ramain P: **Transcriptional activity of pannier is regulated negatively by heterodimerization of the GATA DNA-binding domain with a cofactor encoded by the u-shaped gene of Drosophila.** *Genes & development* 1997, **11**(22):3096–3108, [[http://www.ncbi.nlm.nih.gov/pubmed/9367990]].

[52] Hassan B, Li L, Bremer KA, Chang W, Pinsonneault J, Vaessin H: **Prospero is a panneural transcription factor that modulates homeodomain protein activity.** *Proceedings of the National Academy of Sciences of the United States of America* 1997, **94**(20):10991–10996, [[http://www.ncbi.nlm.nih.gov/pubmed/9380747]].

[53] Ren B, Maniatis T: **Regulation of Drosophila Adh promoter switching by an initiator-targeted repression mechanism.** *The EMBO journal* 1998, **17**(4):1076–1086, [[http://www.ncbi.nlm.nih.gov/pubmed/9463385]].

[54] Taylor HS: **A regulatory element of the empty spiracles homeobox gene is composed of three distinct conserved regions that bind regulatory proteins.** *Molecular reproduction and development* 1998, **49**(3):246–253, [[http://www.ncbi.nlm.nih.gov/pubmed/9491376]].

[55] Vögtli M, Elke C, Imhof MO, Lezzi M: **High level transactivation by the ecdysone receptor complex at the core recognition motif.** *Nucleic acids research* 1998, **26**(10):2407–2414, [[http://www.ncbi.nlm.nih.gov/pubmed/9580693]].

[56] Jun S, Wallen RV, Goriely A, Kalionis B, Desplan C: **Lune/eye gone, a Pax-like protein, uses a partial paired domain and a homeodomain for DNA recognition.** *Proceedings of the National Academy of Sciences of the United States of America* 1998, **95**(23):13720–13725, [[http://www.ncbi.nlm.nih.gov/pubmed/9811867]].
